# The Mechanosensitive PIEZO1 Channel Contributes to the Reaction of RAW264.7 Macrophages to Mechanical Strain

**DOI:** 10.1155/mi/9998838

**Published:** 2025-05-18

**Authors:** Agnes Schröder, Hanna Engelhardt, Andressa Nogueira, Björn E. Clausen, Christian Kirschneck, Jonathan Jantsch, Peter Proff, Kathrin Renner, Eva Paddenberg-Schubert

**Affiliations:** ^1^Department of Orthodontics, University Hospital Regensburg, Regensburg, Germany; ^2^Institute for Medical Microbiology and Hygiene, University Regensburg, Regensburg, Germany; ^3^Department of Periodontology and Operative Dentistry, University Medical Center Mainz, Mainz, Germany; ^4^Institute for Molecular Medicine, University Medical Center Mainz, Mainz, Germany; ^5^Department of Orthodontics, University Hospital Bonn, Bonn, Germany; ^6^Institute for Medical Microbiology, Immunology and Hygiene, University Hospital Cologne and Faculty of Medicine, University of Cologne, Cologne, Germany; ^7^Department of Otorhinolaryngology, University Hospital Regensburg, Regensburg, Germany

**Keywords:** macrophages, mechanical strain, orthodontic tooth movement, PIEZO1

## Abstract

The mechanosensitive channel ‘piezo type mechanosensitive ion channel component 1' (PIEZO1) plays a regulatory role in the response of periodontal ligament fibroblasts (PDLFs) to the mechanical strain that occurs during orthodontic tooth movement. In addition to PDLFs, immune cells such as macrophages are also exposed to mechanical stimuli. Macrophages respond to mechanical strain with increased expression of inflammatory mediators. The role of PIEZO1 in this response remains elusive. To investigate the effect of PIEZO1 activation, RAW264.7 macrophages were stimulated with the PIEZO1 activator YODA1 without concurrent application of pressure. To further examine the specific role of PIEZO1 during mechanical strain, RAW264.7 macrophages were exposed to mechanical strain without and with simultaneous inhibition of PIEZO1 either by chemical inhibition (GsMTx4) or siRNA silencing. The expression of genes and proteins involved in orthodontic tooth movement was examined by quantitative PCR, western blot, and enzyme-linked immunosorbent assay (ELISA). Activation of PIEZO1 by YODA1 or mechanical strain increased the expression of inflammatory cytokines and osteoprotegerin (Opg), which is critically involved in bone remodeling processes. Conversely, inhibition of the PIEZO1 channel attenuates the effects of mechanical stress. In conclusion, our data demonstrate that the PIEZO1 channel is a major contributor to the response of macrophages to mechanical strain encountered during orthodontic tooth movement and affects the expression of inflammatory and bone remodeling factors.

## 1. Introduction

Orthodontic tooth movement is a process that combines catabolic and anabolic responses to externally applied mechanical forces, resulting in a physiological adaptation of the alveolar bone to mechanical stress [1]. Application of orthodontic force creates zones of pressure and tension in the periodontal ligament [2, 3]. Bone resorption occurs in pressure zones and bone apposition in tension zones. A prerequisite for this remodeling activity is a sterile inflammatory process [4]. In addition to fibroblasts, the periodontal ligament includes immune cells, like T cells or macrophages [5–7].

Macrophages naturally reside in tissues throughout the body or are recruited to tissues during inflammatory processes [8, 9]. They have a critical function in surveillance of pathogens, promotion of inflammation, wound healing, and regeneration [8, 10, 11]. This functional diversity results from the ability of macrophages to dynamically respond and to adapt to signals from the local microenvironment [8, 11]. In the early stages of orthodontic tooth movement, macrophages are recruited to the site of the sterile inflammation [7]. Various inflammatory factors promote polarization to classically activated macrophages on the pressure side of the periodontal ligament in response to orthodontic tooth movement [7, 12]. This is primarily mediated by periodontal ligament fibroblasts (PDLFs) by releasing proinflammatory enzymes, cytokines, and chemokines, but can be modulated by macrophages [13, 14]. Classically activated macrophages produce ‘tumor necrosis factor' (TNF), accelerating orthodontic tooth movement [7, 12]. Furthermore, macrophages are capable of producing cytokines such as ‘interleukin-1beta' (IL1*β*) and ‘interleukin-6' (IL6), whose expression is known to increase during orthodontic tooth movement [4, 15].

The ion channel ‘piezo type mechanosensitive ion channel component 1' (PIEZO1) appears to play a critical role in converting mechanical stimuli into intracellular molecular processes, in the form of electrochemical signals [5, 16–18] and was shown to be essential for alveolar bone remodeling during orthodontic treatment in a rat model [19]. Cells respond to mechanical stress by opening mechanosensitive ion channels [20]. Many physiological processes, including touch sensation, cold and heat sensation, proprioception, vascular development, and bone remodeling are mediated by mechanotransduction [18, 21, 22]. Next to mechanical stimuli, expression of PIEZO1 was increased by LPS [23] and during periodontitis [24]. The PIEZO1 channel is a cation-selective ion channel that is permeable to monovalent and divalent cations, such as Na^+^, K^+^, Ca^2+^, and Mg^2+^, with a preference for Ca^2+^ [8, 17, 20, 25]. PIEZO1 is activated by mechanical forces such as shear stress, pressure, and membrane tension, converting these signals into biological signals and triggering a cellular signaling cascade by Ca^2+^ influx through the piezo channels [8, 17, 21, 26, 27]. To date, the structure of the murine PIEZO1 channel has been uncovered [28]. It exhibits a homotrimeric structure that resembles a three-bladed propeller and can be divided into two modules, namely, a peripheral and a central ion-conducting module [17]. The peripheral module consists of three sheet-like helical structures, each composed of 26 transmembrane regions and is connected to the central ion-conducting pore by a bar-like structure [22, 27, 29]. It is currently assumed that mechanotransduction is triggered by a lever-like mechanism [27, 30]. The rod and latch are positioned to transmit conformational changes from the periphery of the propeller blade to the central ion channel [22]. When pressure is applied, the rotor blades stretch and the channel opens [31].

In macrophages, cyclic hydrostatic pressure has previously been shown to involve PIEZO1 in stabilizing hypoxia-inducible factor 1*α* via endothelin-1 to modulate signaling pathways leading to the production of proinflammatory mediators [32]. In addition, PIEZO1 plays an important role in enhancing Toll-like receptor 4 signaling, which involves cytoskeletal rearrangements that affect phagocytosis, ROS production, and antimicrobial activity [23]. In this study, we investigated the impact of PIEZO1 during mechanical strain on RAW264.7 macrophages.

## 2. Material and Methods

### 2.1. Experimental Setups

#### 2.1.1. General Cell Culture Conditions for RAW264.7 Macrophages

RAW264.7 macrophages (400319, Cell Lines Service) were cultured in Dulbecco's modified eagles medium (DMEM High Glucose (D5796, Sigma–Aldrich)) with 10% fetal bovine serum (FBS, P30-3306, PAN Biotech) and 1% antibiotic/antimyotic (AA, A5955, Sigma–Aldrich). For the experiments, cells were transferred to RPMI 1640 with GlutaMax (61870-044, Thermo Scientific) containing 10% FCS and 1% AA. For all experiments 250,000 cells per well of a 24-well plate or 1,000,000 cells per well of a 6-well plate were used.

#### 2.1.2. Setup for Kinetic Experiments With Compressive Strain

In an initial experimental setup RAW264.7 macrophages were compressed for different times (0, 0.5, 1, and 4 h) with a force of 2 g/cm^2^ using sterile zirconium oxide plates after a pre-incubation of 24 h [13, 33].

#### 2.1.3. Experimental Setup for Activation of PIEZO1

YODA1 (5586, Tocris) activates PIEZO1 and promotes the associated cation influx into the cell by shortening the inactive phase of mechanosensitive cation channels [34–36]. After a preincubation period of 24 h, 30 µM YODA1 were added to RAW267.4 macrophages. The same amount of DMSO was pipetted to the control group. After 4 h, the cells were harvested for further processing.

#### 2.1.4. Setup for Inhibition of PIEZO1 in Combination With Compressive Strain

GsMTx4 (4912, Tocris) is an inhibitor of mechanosensitive cation channels and thus also of the PIEZO1 channel [20, 37, 38]. After a 24 h of preincubation period, 2 µM GsMTx4 was added to RAW264.7 macrophages and incubated for an additional hour before pressure application (2 g/cm^2^) [13, 33]. After additional 4 h, the cells were harvested for further processing.

#### 2.1.5. Experimental Setup for PIEZO1 Silencing With siRNA

The experimental setup essential followed earlier published protocols [39]. Two million RAW264.7 macrophages were washed three times with 5 mL OptiMEM (11058-021, Thermo Fisher) and centrifuged for 5 min each time, at 4°C and 300 rpm. OptiMEM was removed and the cell pellet was either treated with 20 µL nonsilencing (ns) siRNA (1027280, Qiagen) or *Piezo1* siRNA (*ΔPiezo1*; L-061455-00-0005, Dharmacon) in combination with 30 µL OptiMEM in precooled electroporation cuvettes (1652088, Bio Rad). The cell suspension was added and made up 50 µL with OptiMEM, resuspended and kept on ice for 5 min. Electroporation was then performed using Gene Pulser Xcell (1652660, Bio-Rad) at 400 V, 150 µF, and 100 Ω for approximately 12–13 ms. After electroporation, 900 µL RPMI without FCS was added to each sample and stored on ice for 10–15 min. Then, 1 mL of RPMI with 20% FCS was added, made up to 6.6 mL with RPMI with 10% FCS and seeded accordingly [39]. Three days after electroporation, compressive strain (2 g/cm^2^) was performed for 4 h. The cells were then harvested for further processing.

### 2.2. RNA Analysis

Essentially, the RNA analysis and was performed as described earlier [13]. Details on RNA isolation, cDNA synthesis and quantitative real-time polymerase chain reaction are given below.

#### 2.2.1. RNA Isolation

RNA extraction was performed using RNA Solv Reagent (R6830-01, VWR). The supernatant was discarded and 250 µL RNA Solv reagent was pipetted onto the cells and the lysed cells were transferred to a tube. After addition of 100 µL chloroform (Fi 10122190, Fisher Chemicals), each sample was vortexed for 30 s and then incubated on ice for 15 min before centrifugation at 4°C and 13,000rpm for 15 min. The aqueous supernatant was mixed with 500 µL isopropanol (20,842,330, VWR) and incubated at −80°C overnight. Samples were centrifuged for 30 min at 4°C and 13,000 rpm. The supernatant was carefully removed and the pellet was washed by adding 750 µL of 80% ethanol (51976, Sigma–Aldrich) in ultrapure water (L0015, Biochrom), centrifuged again for 10 min at 4°C and 13,000 rpm. The process was performed twice. The pellet was dried for 30 min, resuspended in 20 µL RNase free water (T143, Carl Roth), and RNA concentration was measured in the NanoPhotometer (N60, Implen).

#### 2.2.2. cDNA Synthesis

Equal RNA concentrations in a volume of 5.5 µL were mixed with 4.5 µL master mix composed of 2 µL MMLV buffer (M531A, Promega), 0.5 µL oligo dT (SO 132, Thermo Fisher), 0.5 µL random hexamer (SO142, Thermo Fisher), 0.5µL 10 mM dNTP's (L785.2, Carl Roth), 0.5 µL RNase inhibitor (EO0382, Thermo Fisher), and 0.5 µL M-MLV reverse transcriptase (M1705, Promega). Samples were placed in a thermal cycler (Thermocycler Tone 96G, Biometra) and were heated to 37°C for 1 h and 95°C for 2 min.

#### 2.2.3. Quantitative Real-Time Polymerase Chain Reaction (qPCR)

For qPCR, 1.5 µL of cDNA was pipetted in duplicate onto a 96-well plate and 8.5 µL of primer mix (0.25 µL forward primer (Table 1), 0.25 µL reverse primer (Table 1), 5 µL Luna Universal qPCR mix (M3003E, Biolabs), and 3 µL RNase free water) was added. Adhesive optical film (712350, Biozym Scientific GmbH) was applied over the plate. The 96-well plate was briefly centrifuged, placed in the thermal cycler (Mastercycler ep realplex, Eppendorf) and the program was started (95°C for 2 min, 45 cycles of 10 s each at 95°C, 20 s at 60°C, and 8 s at 72°C, with a final melting curve). A combination of *Eef1a1*and *Sdha* was used as reference genes (Table 1). Relative gene expression was calculated using the 2^−*Δ*CT^ method with *Δ*CT as difference of the CT value of the target gene and the geometric mean of *Eef1a1/Sdha* [40, 41].

### 2.3. Western Blot Analysis

Essentially, immunoblotting was performed as described earlier [42]. Cells were lysed with 100 µL of CelLytic (C2978, Sigma Aldrich) supplemented with 1 µL proteinase inhibitor (87786, Thermo Fisher Scientific) and protein concentration was assessed using RotiQuant (K015.3, Carl Roth) following manufacturer's instructions. Equal amounts of protein were loaded on 8% polyacrylamide gels and transferred to polyvinylidenfluoride membranes (T830.1, Carl Roth). The membranes were blocked in 5% milk (T145.3, Carl Roth) in tris-buffered saline with Tween20 (TBS-T) for 1 h at room temperature. The membranes incubated in the primary antibodies PIEZO1 (MBS7602668, mybiosource) and ACTIN (E1C602-2, EnoGene) were overnight at 4°C. The membranes were washed three times in TBS-T and then incubated for 1 h at room temperature in the secondary antibody (611-1302, Rockland Immunochemicals). Afterwards they were again washed three times in TBS-T. The antibody exposed membranes were then exposed to Luminata Crescendo Western HRP Substrate (WBLUR0100, Sigma Aldrich) and the signal was digitized using the VWR Genoplex documentation system (VWR international).

### 2.4. Enzyme-Linked Immunosorbent Assay (ELISA)

The supernatants were collected and stored at −20°C until the ELISA was performed. Prior to ELISA measurements, supernatants were thawed and stored on ice 30 min before the start of the experiment. Each ELISA was performed according to the manufacturer's instructions and with a kit specific for the respective cytokines and mediators (IL6 (1311639521, Boster Bio), prostaglandin E2 (PGE2; 514010, Cayman), osteoprotegerin (OPG; 78092220, Thermo Fisher Scientific), and TNF (MBS335449, MyBioSource)).

### 2.5. Statistics

Statistical analysis was performed using the GraphPadPrism program (version 9). Prior to statistical analysis, all absolute data values were divided by the respective arithmetic mean of the control group without mechanical loading to normalize the data values to the controls. Each symbol represents a data value, the horizontal lines show the mean and the vertical lines show the standard error. Normal distribution of the data was examined with the Shapiro–Wilk test and homogeneity with the Brown–Forsythe test. Welch-corrected ANOVAs with Dunnett's T3 post hoc tests were performed.

## 3. Results

### 3.1. Mechanical Strain Increased Expression of the PIEZO1 Channel and Inflammatory and Bone Remodeling Genes

**Figure 1:**
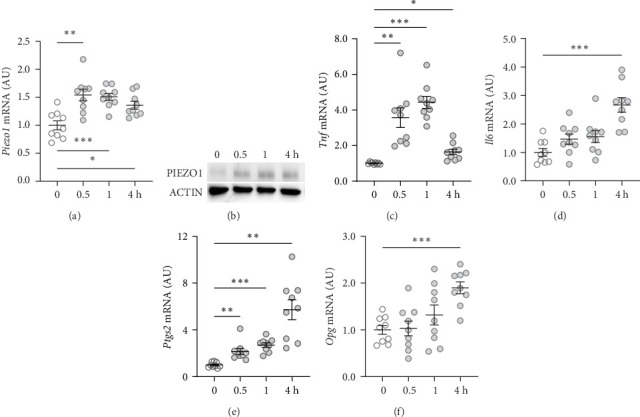
Mechanical strain increased the expression of the PIEZO1 channel and inflammatory and bone remodeling genes. (a) Gene and (b) protein expression of PIEZO1 as well as gene expression of (c) tumor necrosis factor alpha (*Tnf*), (d) interleukin-6 (*Il6*), (e) prostaglandin endoperoxide synthase-2 (*Ptgs2*), and (f) osteoprotegerin (*Opg*). *n* = 9; statistics: Welch-corrected ANOVAs were performed with Dunnett's T3 post hoc tests; *⁣*^*∗*^*p* < 0.05; *⁣*^*∗∗*^*p* < 0.01; *⁣*^*∗∗∗*^*p* < 0.001.

First, the impact of compressive strain on PIEZO1 gene and protein expression was tested. Gene expression of *Piezo1* was upregulated as early as 0.5 h after pressure application and remained elevated throughout the study period as determined by quantitative PCR (*p* ≤ 0.011; Figure 1a) and by western blot (Figure 1b). Next, gene expression of the inflammatory mediators *Tnf*, *Il6*, and ‘prostaglandin endoperoxide synthase-2' (*Ptgs2*) was investigated after different compressive force periods. *Tnf* mRNA was increased after 0.5 h of pressure application and remained stably upregulated throughout the study time span (*p* ≤ 0.011; Figure 1c). Gene expression of *Il6* was significantly increased only after 4 h of compressive strain (*p* < 0.001; Figure 1d). Like *Tnf*, *Ptgs2* was already twofold elevated after 0.5 h (*p*=0.006), but showed even a fivefold increase after 4 h (*p*=0.002; Figure 1e). Similar to *Il6*, *Opg* mRNA was significantly upregulated only after 4 h of pressure application (*p* < 0.001; Figure 1f). Since all genes investigated were significantly elevated after 4 h of compressive strain, this period was chosen for the following experiments.

### 3.2. Chemical Activation of PIEZO1 Promotes the Expression of Inflammatory and Bone Remodeling Genes

**Figure 2:**
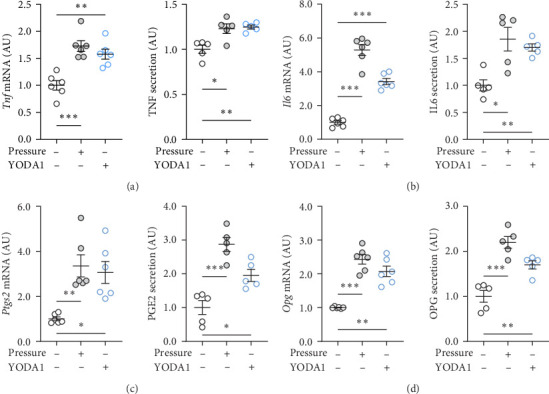
Activation of PIEZO1 by YODA1 enhanced inflammatory and bone remodeling gene expression. Gene expression and protein secretion of (a) TNF, (b) IL6, (c) *Ptgs2*/PGE2, and (d) OPG after compressive strain for 4 h or YODA1 treatment. *n* ≥ 5; statistics: Welch-corrected ANOVAs were performed with Dunnett's T3 post hoc tests; *⁣*^*∗*^*p* < 0.05; *⁣*^*∗∗*^*p* < 0.01; *⁣*^*∗∗∗*^*p* < 0.001.

PIEZO1 was chemically activated with YODA1 in RAW264.7 macrophages and compared with the effects of compressive strain. TNF gene and protein expression were increased after compressive strain (*p* ≤ 0.022; Figure 2a) as well as treatment with YODA1 (*p* ≤ 0.005; Figure 2a). Similarly, compressive strain for 4 h (*p* ≤ 0.023; Figure 2b) and application of YODA1 (*p* ≤ 0.002; Figure 2b) increased IL6 on mRNA and protein level. The same effects were visible in RAW264.7 macrophages for the inflammatory gene *Ptgs2* and its product PGE2 (Figure 2c). Likewise, gene expression and protein secretion of OPG were increased by both compressive strain (*p* < 0.001) and YODA1 treatment (*p* ≤ 0.006; Figure 2d). These data show that activation of the PIEZO1 ion channel alone induces inflammatory macrophage activation similar to that induced by compression.

### 3.3. Chemical Inhibition of PIEZO1 During Compressive Force Mitigates Expression of Inflammatory and Bone Remodeling Genes

**Figure 3:**
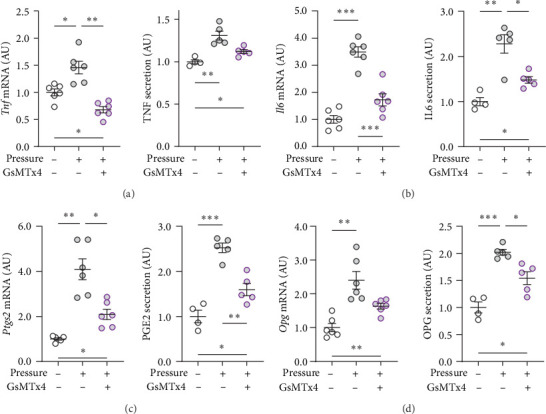
Inhibition of the PIEZO1 channel by GsMTx4 impairs the effects of mechanical strain. Gene and protein secretion of (a) TNF, (b) IL6, (c) PTGS2/PGE2 and (d) OPG after compressive strain for 4 h without or with additional GsMTx4 treatment. *n* ≥ 4; statistics: Welch-corrected ANOVAs were performed with Dunnett's T3 post hoc tests; *⁣*^*∗*^*p* < 0.05; *⁣*^*∗∗*^*p* < 0.01; *⁣*^*∗∗∗*^*p* < 0.001.

GsMTx4 is an inhibitor of several mechanosensitive ion channels, including PIEZO1 [43]. GsMTx4 was added to cell cultures while simultaneously a compressive stress was applied. As *Piezo1* deficiency does not impact cytokine release in untreated cells [8], we focused on the role of mechanosensitive channels in cells exposed only to mechanical stress. The pressure-induced increase in *Tnf* gene expression was inhibited by GsMTx4 (*p*=0.002; Figure 3a), even below the *Tnf* mRNA levels of the uncompressed control group (*p*=0.011). Treatment with GsMTx4 also tended to reduce TNF secretion after pressure (*p*=0.053), but there was still a significant, albeit reduced, and effect of pressure on TNF secretion (*p*=0.022; Figure 3a). GsMTx4 treatment significantly reduced the elevated gene expression and protein secretion of IL6 after mechanical strain (*p* ≤ 0.038; Figure 3b). As with TNF, the pressure-induced increase in IL6 secretion was reduced, although not completely inhibited, by GsMTx4 treatment (*p*=0.017; Figure 3b). Pressure-induced *Ptgs2* gene expression and PGE2 secretion were suppressed by GsMTx4 (*p* ≤ 0.017; Figure 3c), but again a diminished residual effect remained even after inhibition (*p* ≤ 0.049).

For *Opg* mRNA expression, a clear pressure effect was detected (*p* ≤ 0.006), which was only slightly reduced with GsMTx4 (*p*=0.080; Figure 3d), while the increased secretion of OPG protein with pressure (*p* ≤ 0.029) was significantly reduced in the presence of GsMTx4 (*p*=0.027; Figure 3d). Taken together, these data suggest that mechanosensitve ion channels like PIEZO1 are key contributors to pressure-induced macrophage activation.

### 3.4. PIEZO1 mRNA Silencing Impairs Compressive Force-Induced Gene Expression for Inflammation and Bone Remodeling

**Figure 4:**
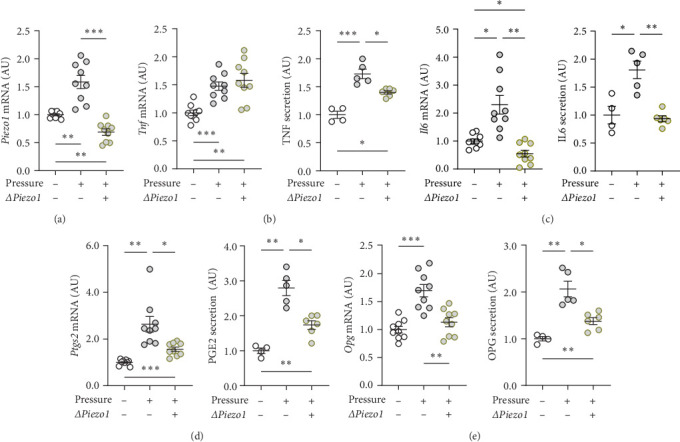
Silencing of the PIEZO1 channel by siRNA influence the effects of mechanical strain. Silencing of *Piezo1* gene expression was evaluated by qPCR (a). Gene expression and protein secretion of (b) TNF, (c) IL6, (d) PTGS2/PGE2, and (e) OPG after compressive strain for 4 h in control ns siRNA (*ΔPiezo1*:−) or *Piezo1*-siRNA (*ΔPiezo1*:+) treated RAW264.7 macrophages. *n* ≥ 4; statistics: Welch-corrected ANOVAs were performed with Dunnett's T3 post hoc tests; *⁣*^*∗*^*p* < 0.05; *⁣*^*∗∗*^*p* < 0.01; *⁣*^*∗∗∗*^*p* < 0.001.

To inhibit PIEZO1 function, *Piezo1* silencing using *Piezo1*-specific siRNA was performed. Accordingly, *Piezo1* mRNA expression was reduced after treatment with *Piezo1*-specific siRNA compared to cells treated with ns RNA (*p*=0.003; Figure 4a). Although pressure-induced *Tnf* gene expression was not changed by *Piezo1* silencing (*p*=0.850; Figure 4b), there was a slight but significant reduction of TNF secretion after *Piezo1* silencing (*p*=0.037; Figure 4b). Of note, TNF secretion after *Piezo1* silencing was significantly higher than in macrophages treated with ns RNA (*p*=0.012), while *Piezo1* silencing did not affect *Tnf* mRNA expression. These findings suggested that Piezo1 contributes to TNF release in a posttranslational manner.

Treatment with *Piezo1* siRNA reduced *Il6* mRNA expression compared to ns siRNA-treated conditions without (*p*=0.020) and with compressive strain (*p*=0.002; Figure 4b), while IL6 protein was only suppressed compared to the pressure-induced IL6 secretion (*p*=0.008; Figure 4b). *Ptgs2* mRNA expression and PGE2 secretion were elevated after compressive strain with ns siRNA (*p* ≤ 0.004). Pressure-induced *Ptgs2* expression on mRNA (*p* < 0.001) protein (*p*=0.003) level remained significantly higher after *Piezo1* siRNA treatment than in the absence of pressure (Figure 4c), but there was still significant inhibition of pressure-induced *Ptgs2* expression in *Piezo1* silenced cells (*p* ≤ 0.036; Figure 4c). Likewise, *Piezo1* siRNA treatment reduced (*p* ≤ 0.026), but did not abolish the effect of pressure application on *Opg* mRNA (*p*=0.008; Figure 4d). These data demonstrate that silencing of *Piezo1* mRNA inhibits pressure-mediated macrophage activation, confirming the results obtained with blockade of the PIEZO1 ion channel.

## 4. Discussion

Macrophages play a critical role in promoting tissue inflammation and regeneration [8, 11] and are recruited to pressure zones in early stages of orthodontic tooth movement [5]. The ion channel PIEZO1 converts mechanical stimuli into intracellular molecular processes in the form of electrochemical signals [16, 17, 44]. The present study investigated the impact of PIEZO1 on the activation of macrophages after mechanical strain that occurs in the pressure zone of the periodontal ligament during orthodontic treatment. Consistent with our data, PIEZO1 has previously been shown to promote an ”M1-like” inflammatory phenotype marker by increasing TNF and IL6 expression upon stimulation [8, 45]. PIEZO1 is able to modulate the function of PDLF [42, 46]. Like RAW264.7 macrophages, PDLF respond with increased PIEZO1 expression after a short period of static compression. In PDLF, PIEZO1 has been implicated to modulate the expression of inflammatory genes and bone remodeling processes by affecting OPG expression [42, 46]. As a decoy receptor of the receptor activator of NF*κ*B ligand (RANKL), OPG inhibits osteoclastogenesis, and osteoclast activity [47, 48], while RANKL promotes osteoclast activation, leading to increased bone loss [4, 47]. In a model of rapid maxillary expansion, PIEZO1 was shown to be associated with bone remodeling by and osteogenesis of periostal-derived stem cells [49].

Macrophages may play an additional role in modulating bone metabolism by producing cytokines and factors that can influence osteoblast and osteoclast activity [50, 51]. Here we demonstrate that RAW264.7 macrophages respond to mechanical strain by upregulating OPG, suggesting that this might prevent excessive alveolar bone resorption.

Activation of PIEZO1 with YODA1 occurs through attachment to an allosteric binding pocket located in close proximity to its central pore-forming unit [52]. Syeda et al. [53] reported that YODA1 affects the sensitivity and inactivation kinetics of mechanically triggered responses, which may lead to the activation of the PIEZO1 channel. We show that activation of PIEZO1 by YODA1 results in a significant increase in proinflammatory cytokines and OPG, suggesting that the PIEZO1 channel exerts a significant influence on the expression of these factors. In line with this, inhibition of PIEZO1 with GsMTx4 leads to a reduction in OPG and IL6 expression, which is also observed after PIEZO1 mRNA silencing. This suggests that PIEZO1 is involved in inducing inflammatory responses under mechanical stress.

Our data demonstrate that after compressive strain in RAW264.7 macrophages, there is a concomitant increase in PIEZO1 expression, secretion of inflammatory mediators, and OPG. As already described, the inflammatory mediators TNF, IL6, and PGE2 are increased after mechanical stress [13, 54]. These soluble factors are important and multifaceted regulators of the immune responses. Pharmacological inhibition of mechanotransduction with GsMTx4, which, in addition to PIEZO1, inhibits cationic mechanosensitive channels [43], reduced *Tnf* expression, and TNF release in the supernatant. Specific targeting of Piezo1 by deletion of *Piezo1* reduced compression-induced TNF release, while *Tnf* gene expression was not altered in *Piezo1*-deficient cells. This indicates that PIEZO1 specifically interferes with TNF release upon compression.

TNF-*α* converting enzyme (TACE), also referred to as ADAM17, has been demonstrated to play a pivotal role in the process of TNF release from macrophages [55, 56]. TACE is critical for cleaving of membrane-bound TNF, which is initially produced as a transmembrane protein [56]. For soluble TNF to be released, the transmembrane protein must be cleaved from the cell surface by TACE [56]. The regulatory mechanisms governing TACE activity are complex and not yet fully elucidated [56]. Our data suggest that *Piezo1*-dependent signaling may play a role in this context. There is evidence that Piezo1 is able to modify the activity of TACE/ADAM17 in human epithelial cell lines [57]. Therefore, it is tempting to speculate that *Piezo1* deletion may also alter the proteolytic activity of this proteinase, and thus, the cleavage of TNF in macrophages.

TNF increases the production of other inflammatory mediators, promotes the migration of immune cells to sites of inflammation, and activates macrophages [58, 59]. Similarly, IL6 is released in response to inflammation, infection, and injury. However, IL6 can both stimulate and dampen the immune response [60, 61]. During inflammatory processes, the expression of PTGS2 is increased as well. This enzyme catalyzes the conversion of arachidonic acid to PGE2 [62]. PGE2, in turn, functions as a crucial mediator in inflammatory processes and pain mediation in the body [63, 64] as well as during orthodontic tooth movement [14].

Jiang et al. [65] investigate the expression pattern of PIEZO1 after orthodontic tooth movement in a rat model. The expression of PIEZO1 was increased in cells present in the periodontal ligament by orthodontic forces. They demonstrate that the PIEZO1 channel plays a critical role in bone remodeling processes needed to achieve orthodontic tooth movement by promoting osteogenesis and osteoclastic activities [65].

In line with this, our study demonstrates that the PIEZO1 channel mediates the reaction of RAW264.7 macrophages to mechanical strain occurring during orthodontic tooth movement, affecting expression of inflammatory cytokines and the bone-remodeling factor OPG. These findings may serve as the basis for therapeutic approaches that specifically target the PIEZO1 channel to shape alveolar bone remodeling and inflammation during tooth movement in a stable and controlled manner.

To the best of our knowledge, this is the first study, investigating the role of PIEZO1 on the reaction of RAW264.7 macrophages to mechanical compression. This complements studies on the analysis of the role of PIEZO1 in cyclic stress in bone marrow-derived macrophages [10]. It should be noted that the experiments in this study were performed in vitro to demonstrate the fundamental effects of PIEZO1 activation or inhibition on macrophages upon exposure to mechanical compression. However, this simplified approach cannot reproduce the complexity of the in vivo mechanisms in the human organism. Moreover, it is likely that other molecules and signal transduction pathways such as cell–cell junction proteins are involved in relaying mechanic stress to macrophages [66]. For instance, E-cadherin/catenin complex which play an important role in in mononuclear phagocytes such as Langerhans cells and fusion of alternatively activated macrophages could be involved here as well [67, 68].

In conclusion, mechanical stress upregulates PIEZO1 expression and triggers inflammatory responses in RAW264.7 macrophages. Activation of PIEZO1 without concomitant pressure loading leads to an enhanced inflammatory response and OPG expression. In contrast, inhibition of PIEZO1 reduces inflammatory responses and OPG expression upon mechanical stimulation. Overall, our results suggest that PIEZO1 plays an important role in macrophage response to mechanical stress.

## Figures and Tables

**Table 1:** Alphabetical list of the reference and target gene primers used for pPCR.

Gene	Gene name	Forward primer	Reverse primer
*Eef1a1*	Eukaryotic translation elongation factor-1-alpha-1	AAAACATGATTACAGGCACATCCC	GCCCGTTCTTGGAGATACCAG
*Il6*	Interleukin 6	AAAGCCAGAGTCCTTCAGAGAG	CCTTAGCCACTCCTTCTGTGAC
*Opg*	Osteoprotegerin	CCTTGCCCTGACCACTCTTAT	CACACACTCGGTTGTGGGT
*Ptgs2*	Prostaglandin–endoperoxide synthase 2	TCCCTGAAGCCGTACACATC	TCCCCAAAGATAGCATCTGGAC
*Sdha*	Succinate dehydrogenase complex, subunit A	AACACTGGAGGAAGCACACC	AGTAGGAGCGGATAGCAGGAG
*Tnf*	Tumor necrosis factor	ACAAGCCTGTAGCCCACGTC	TTGTTGTCTTTGAGATCCATGCC

## Data Availability

The data are available on request from the authors.
